# Anticancer activity of biogenerated silver nanoparticles: an integrated proteomic investigation

**DOI:** 10.18632/oncotarget.23859

**Published:** 2017-12-23

**Authors:** Miriam Buttacavoli, Nadia Ninfa Albanese, Gianluca Di Cara, Rosa Alduina, Claudia Faleri, Michele Gallo, Giuseppe Pizzolanti, Giuseppe Gallo, Salvatore Feo, Franco Baldi, Patrizia Cancemi

**Affiliations:** ^1^ Department of Biological Chemical and Pharmaceutical Sciences and Technologies (STEBICEF), University of Palermo, Palermo, Italy; ^2^ Center of Experimental Oncobiology (C.OB.S.), La Maddalena Hospital III Level Oncological Dept., Palermo, Italy; ^3^ Department of Life Science, University of Siena, Siena, Italy; ^4^ Department of Molecular Science and Nanosystems, Cà Foscari University of Venice, Venice, Italy; ^5^ Biomedical Department of Internal and Specialist Medicine (DIBIMIS), Section of Endocrinology, University of Palermo, Palermo, Italy; ^6^ Advanced Technologies Network Center (ATeN), University of Palermo, Palermo, Italy

**Keywords:** silver nanoparticles (AgNPs), bacteria, breast cancer cells, anticancer activity, proteomics

## Abstract

Silver nanoparticles (AgNPs), embedded into a specific polysaccharide (EPS), were biogenerated by *Klebsiella oxytoca* DSM 29614 under aerobic (AgNPs-EPS^aer^) and anaerobic conditions (AgNPs-EPS^anaer^). Both AgNPs-EPS matrices were tested by MTT assay for cytotoxic activity against human breast (SKBR3 and 8701-BC) and colon (HT-29, HCT 116 and Caco-2) cancer cell lines, revealing AgNPs-EPS^aer^ as the most active, in terms of IC50, with a more pronounced efficacy against breast cancer cell lines. Therefore, colony forming capability, morphological changes, generation of reactive oxygen species (ROS), induction of apoptosis and autophagy, inhibition of migratory and invasive capabilities and proteomic changes were investigated using SKBR3 breast cancer cells with the aim to elucidate AgNPs-EPS^aer^ mode of action. In particular, AgNPs-EPS^aer^ induced a significant decrease of cell motility and MMP-2 and MMP-9 activity and a significant increase of ROS generation, which, in turn, supported cell death mainly through autophagy and in a minor extend through apoptosis. Consistently, TEM micrographs and the determination of total silver in subcellular fractions indicated that the Ag^+^ accumulated preferentially in mitochondria and in smaller concentrations in nucleus, where interact with DNA. Interestingly, these evidences were confirmed by a differential proteomic analysis that highlighted important pathways involved in AgNPs-EPS^aer^ toxicity, including endoplasmic reticulum stress, oxidative stress and mitochondrial impairment triggering cell death trough apoptosis and/or autophagy activation.

## INTRODUCTION

Cancer is a multifaceted disease, extremely variable in its presentation, development and outcome. It is well established that cancer is a multifactorial disease caused by a complex mixture of genetic and environmental factors. However the knowledge of the genetic, molecular, and cellular basis of cancer can provide new targets and strategies for therapy. Many anticancer drugs are unable to reach their target site in sufficient concentrations and efficiently exert the pharmacological effect without causing irreversible unwanted injury to healthy tissues and cells [1–3]. Nanotechnology offers a wealth of tools to treat cancer by passing biological barriers to deliver therapeutic agents directly [4]. The unique physicochemical characteristics of metal NPs, such as high surface-to-volume ratio, broad optical properties, ease of synthesis and surface functionalization offer new opportunities for cancer therapeutics. The production of silver nanoparticles (AgNPs) has become the object of intense research and several methods have been developed to synthesize noble metal NPs, including physical and chemical ones [5–7].

The biological synthesis of AgNPs have received increasing attention due to the growing need of developing eco-friendly (i. e. “green”) technologies in material synthesis [6,8–12]. Thus, different microorganisms, either bacteria or fungi, have been used as potential cell-factories for both intra and extra cellular production of AgNPs [9, 13]. The mechanism of biological formation of metal-NPs is mainly due to the capability of biopolimers and, in particular, microbial exopolysaccharides (EPS) to act as metal reducers and/or stabilizers [14–16]. The *Klebsiella oxytoca* DSM 29614 EPS is composed of a branched heptasaccharide repeating unit (1 galactose, 4 rhamnoses, 2 glucuronic acids), with metal binding properties during the fermentative biosynthetic process [17, 18]. *K. oxytoca* strains are ubiquitous bacteria mainly studied as opportunistic pathogens which are responsible for nosocomial infections [19]. However, many *K. oxytoca* strains are also known to produce exopolysaccharides of environmental and pharmaceutical interest [17, 20]. Furthermore, the production of other metal nanoparticles embedded in this EPS has already been reported [21–25].

In this study, we tested the anticancer effect of biogenerated AgNPs-EPS on different cancer cell lines, and then investigated its molecular mechanism of action in the SKBR3 breast cancer cell line. In particular, we found that AgNPs-EPS^aer^ caused: i) a significant decrease of cell viability and motility, ii) an impairment of MMP-2 and MMP-9 activity, and iii) a promotion of ROS generation, which, in turn, induced cell death through apoptosis and autophagy. These evidences were confirmed by a differential proteomic analysis, in which proteomic changes are consistent with the activation of important pathways including endoplasmic reticulum stress, oxidative stress and mitochondrial disfunction triggering cell death trough apoptosis and/or autophagy activation. Finally, TEM micrographs and the determination of total silver in subcellular fractions reinforce the idea that Ag^+^ released from AgNPs-EPS^aer^ firstly in mitochondria and then in nuclei determines cell damage and death.

To the best of our knowledge, this is the first study reporting the mechanism of action of biosynthesized AgNPs through an integrated proteomic approach.

## RESULTS

### Cytotoxic effects of AgNPs-EPS

The cytotoxic effect of AgNPs biogenerated by *Klebsiella oxytoca* DSM 29614 under aerobic (AgNPs-EPS^aer^) and anaerobic conditions (AgNPs-EPS^anaer^) was investigated after 24 h of treatment on two human breast cancer cell lines (SKBR3 and 8701-BC) and three human colon cancer cell lines (HT-29, HCT 116 and Caco-2) by using the MTT assay. The results, expressed as IC_50_ values, calculated from the dose-survival curves, are reported in Table 1. The AgNPs-EPS^aer^ is more active than AgNPs-EPS^anaer^, with SKBR3 and 8701-BC cell lines being more sensitive to AgNPs-EPS^aer^ treatment in comparison to HT-29, HCT 116 and Caco-2 colon cancer cell lines. In particular, SKBR3 cells proliferation was significantly inhibited by AgNPs-EPS^aer^ with an IC_50_ value of 5 μg/ml, while an IC_50_ value of 8 μg/ml was found for 8701-BC cell line. These values were found well within the clinically acceptable concentration of 100 μg/ml [26], suggesting a potential anticancer effect of both biogenerated AgNPs-EPS. Since AgNPs-EPS^aer^ contains more total silver than AgNPs-EPS^anaer^ [24] and the amount of Ag^+^ released from AgNPs-EPS^aer^ is significantly higher than for AgNPs-EPS^anaer^ [25], we believe that the biological activity of AgNPs-EPS is Ag-dependent. None toxic effect was observed for metal-free EPS.

**Table 1:** IC_50_ values at 24 h of Ag-NPs-EPS in selected cancer cell lines

IC_50_ (μg/ml) ± SD			
	AgNPs-EPSanaer	AgNPs-EPSaer	Metal free-EPS
SKBR3	50 ± 1.5	5 ± 0.5	NA
8701-BC	75 ± 1.3	8.2 ± 0.8	NA
HT29	NA	20 ± 2	NA
HCT116	NA	26 ± 2	NA
CaCo2	NA	34 ± 4	NA

Since AgNPs-EPS^aer^ had the highest cytotoxic activity towards breast cancer SKBR3 cells, the selectivity index (SI) was investigated by using a non tumoral mammary epithelial cell line (HB2), as previously reported [27]. Cytotoxic assays were performed for 24h and 48h, and doxorubicin was used as a positive control (Table 2). The SI were quite similar between AgNPs-EPS^aer^ and doxorubicin, a commonly used chemotherapeutic drug [28]. Moreover, this investigation revealed high selectivity for SKBR3 cells [29], with higher SI value for higher treatment time, being SI 3.4 and 5.0 after 24 and 48 h, respectively. Based on these results, SKBR3 cells were further used to study the mechanisms of cytotoxicity associated with the AgNPs-EPS treatments.

**Table 2:** IC_50_ values calculated at 24 and 48 h of AgNPs-EPSaer treatment, in SKBR3 breast cancer cell line and in the non tumoral epithelial mammary cell line HB2

	HB2 IC_50_ ± SD		SKBR3 IC_50_ ± SD		Selectivity index SI (HB2IC_50_/SKBR3 IC_50_)	
	24 h	48 h	24 h	48 h	24 h	48 h
**AgNPsaer**	17 ± 0.5^a^	9 ± 0.8^a^	5 ± 0.5^a^	1.8 ± 0.8^a^	3.4	5
**Doxorubicin**	15.8 ± 2,1^b^	5.3 ± 1^b^	4.1 ± 0.5^b^	1.12 ± 0.1^b^	3.8	4.8

SI was calculated as ratio between AgNPs-EPS^aer^ IC_50_ values obtained for HB2 and SKBR3. ^a^Values expressed as µg/ml; ^b^values expressed as µM.

### Colony formation assay

**Figure 1:**
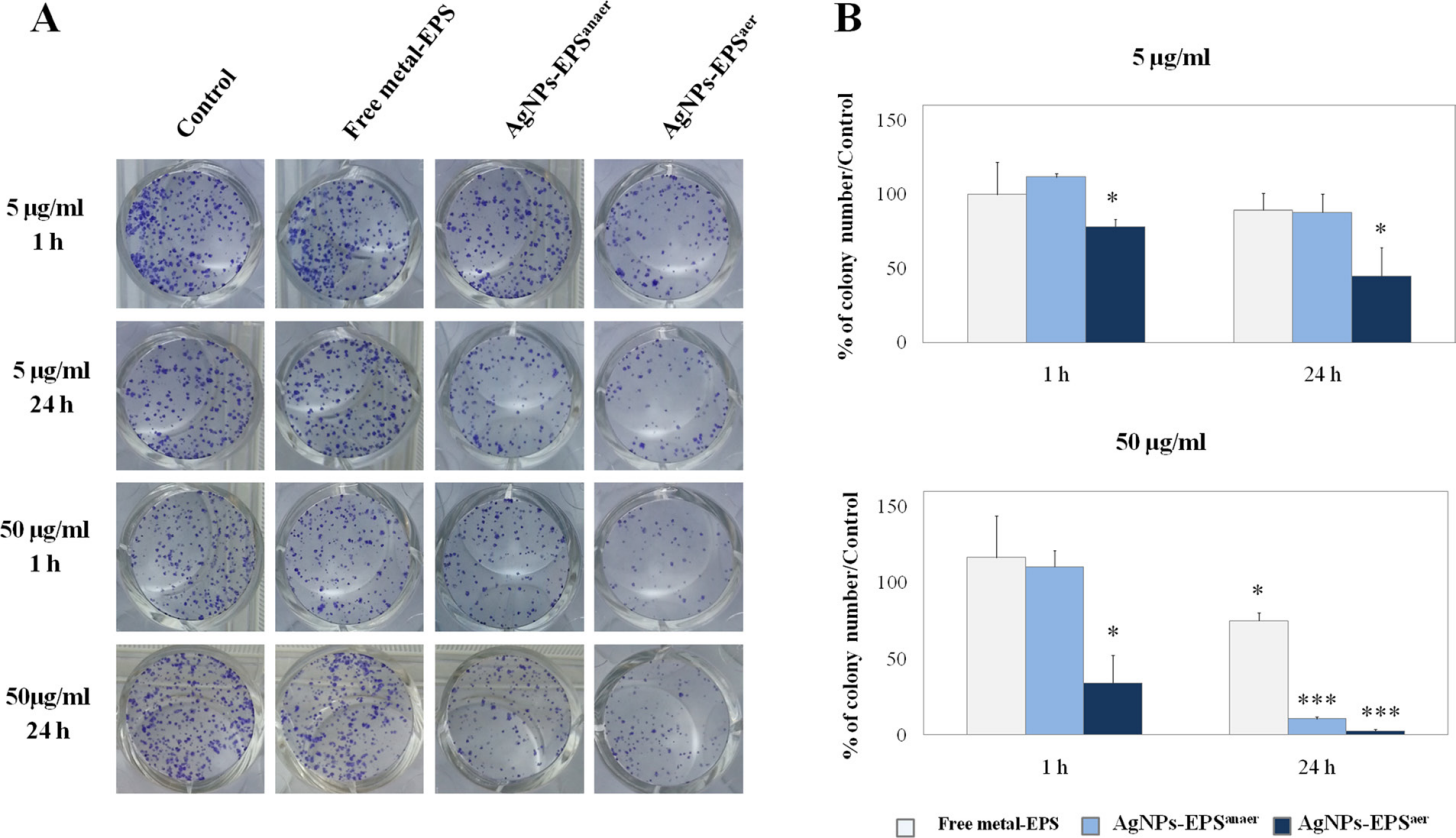
Cytotoxic effect of AgNPs-EPS in SKBR3 cells evaluated by clonogenic assay (**A**) Culture dishes with stained colonies of a representative experiment; (**B**) Statistical results of colony-forming assays presented as percentage of colonies numbers in the respect of untreated cells used as control. Statistical significance was assessed by the Student’s *t*-test: ^*^p < 0.05 was considered significant; ^***^p < 0.001 very highly significant. The data in the graphs are expressed as mean number ± SD of three different experiments.

In order to assess SKBR3 cell viability in terms of reproductive capacity after treatments with the two kinds of AgNPs-EPS, a colony formation assay was performed [30]. To this aim, cells were seeded in appropriate dilutions and treated with two different concentrations (50 and 5 µg/ml) for 1 and 24 h, maintained under normal culture conditions and analysed two weeks later for the formation of colonies. The results (Figure 1) showed that AgNPs-EPS^aer^ treatment inhibited significantly the colony-forming ability of SKBR3 cells in a dose- and time-dependent manner and, interestingly, resulted effective already after 1 h-treatment. On the other hand, AgNPs-EPS^anaer^ treatment inhibited cell proliferation in a dose- and time-dependent manner only at the highest dose of 50 µg/ml. Thus, according MTT assay results, AgNPs-EPS^aer^ exerts a superior biocide activity in comparison to AgNPs-EPS^anaer^. None effect was observed for free-metal EPS.

### AgNPs-EPS inhibit motility and MMPs activity in SKBR3 cells

**Figure 2:**
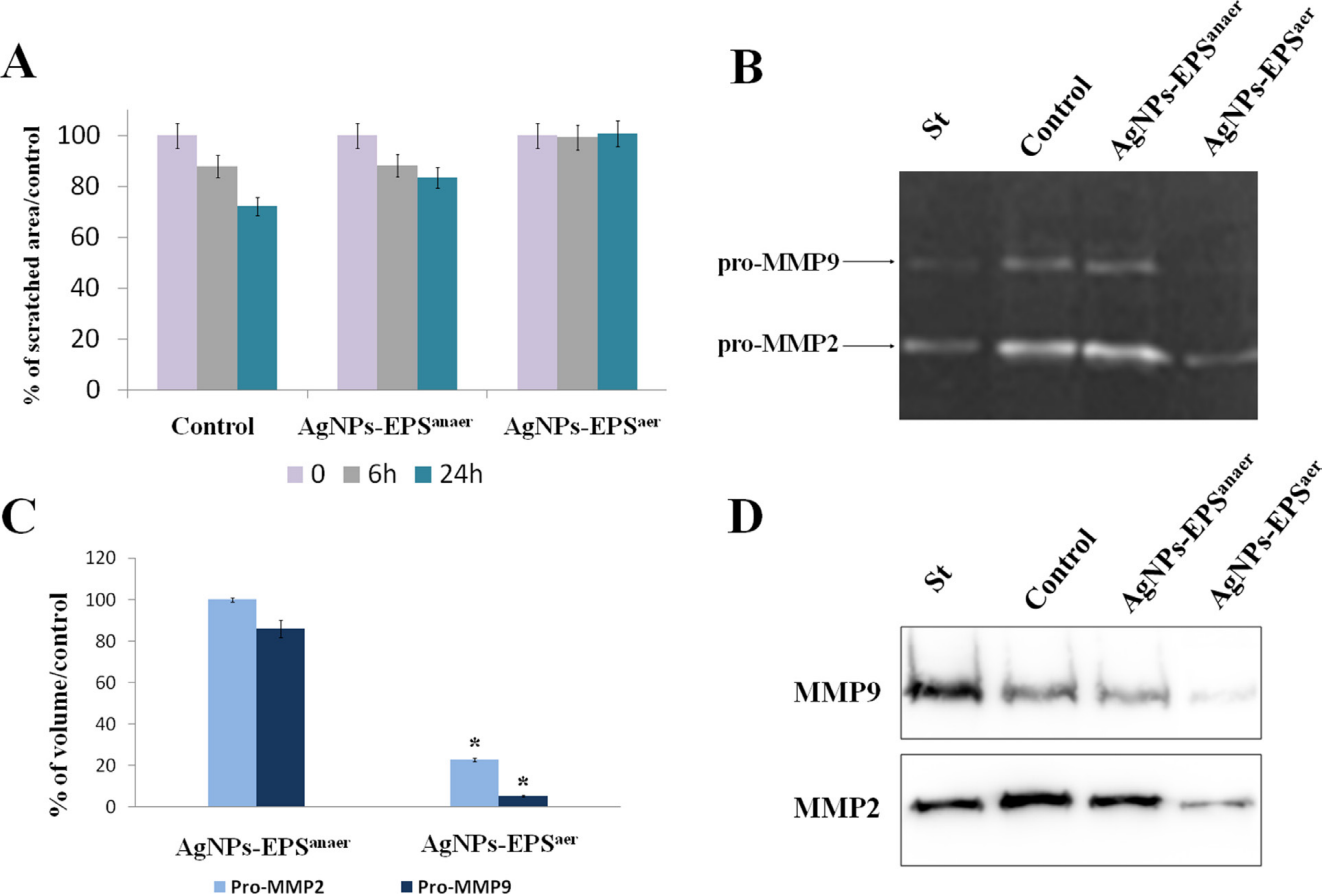
AgNPs-EPS^aer^ inhibit wound healing and MMP9/MMP2 activity in SKBR3 cells (**A**) Inhibitory effect on cell migration assessed by wound healing assay and quantified by measuring the scratched area. (**B**) Zymographic analysis of MMP9 and MMP2 activity in SKBR3 cells. The gelatinolytic bands correspond to the pro MMP9 and pro MMP2. (**C**) Densitometric analyses of proMMP2 and MMP9 secretion. Statistical significance was assessed by the Student’s *t*-test: ^*^p < 0.001. The data in the graphs are expressed as mean of the band-intensity ± SD. (**D**) Western blot validation of MMP9 and MMP2 protein expression in conditioned media.

Migration and invasion represent a cancer progression hallmark [31], so we tested the effect of Ag-NPs-EPS on cell motility by performing a wound-healing assay. Results (Figure 2A) showed an inhibition of migratory ability both at 6 and 24 h in AgNPs-EPS^aer^ treated cells. AgNPs-EPS^anaer^ treatment did not affect significantly the migratory capability of SKBR3 cells. Cell migration represent a complex process that involves the expression of a number of growth factors, cytokines and matrix metalloproteinases (MMPs) [32, 33]. Because of the significant effects induced by AgNPs-EPS^aer^ on cell migration, we evaluated the effects on MMP-2 and MMP-9 enzymatic activities and protein expression in SKBR3 cells using gelatin zymography and western blot. Data revealed a significant decrease of enzymatic activities in AgNPs-EPS^aer^ treated cells with the maximum effect for pro MMP-9 that is almost disappeared. AgNPs-EPS^anaer^ (Figure 2B, 2C and 2D) treatment instead did not affect significantly the activity levels of MMPs, accordingly to the scratch assay results.

### Morphological assessment of SKBR-3 cells treated with both AgNPs-EPS

**Figure 3:**
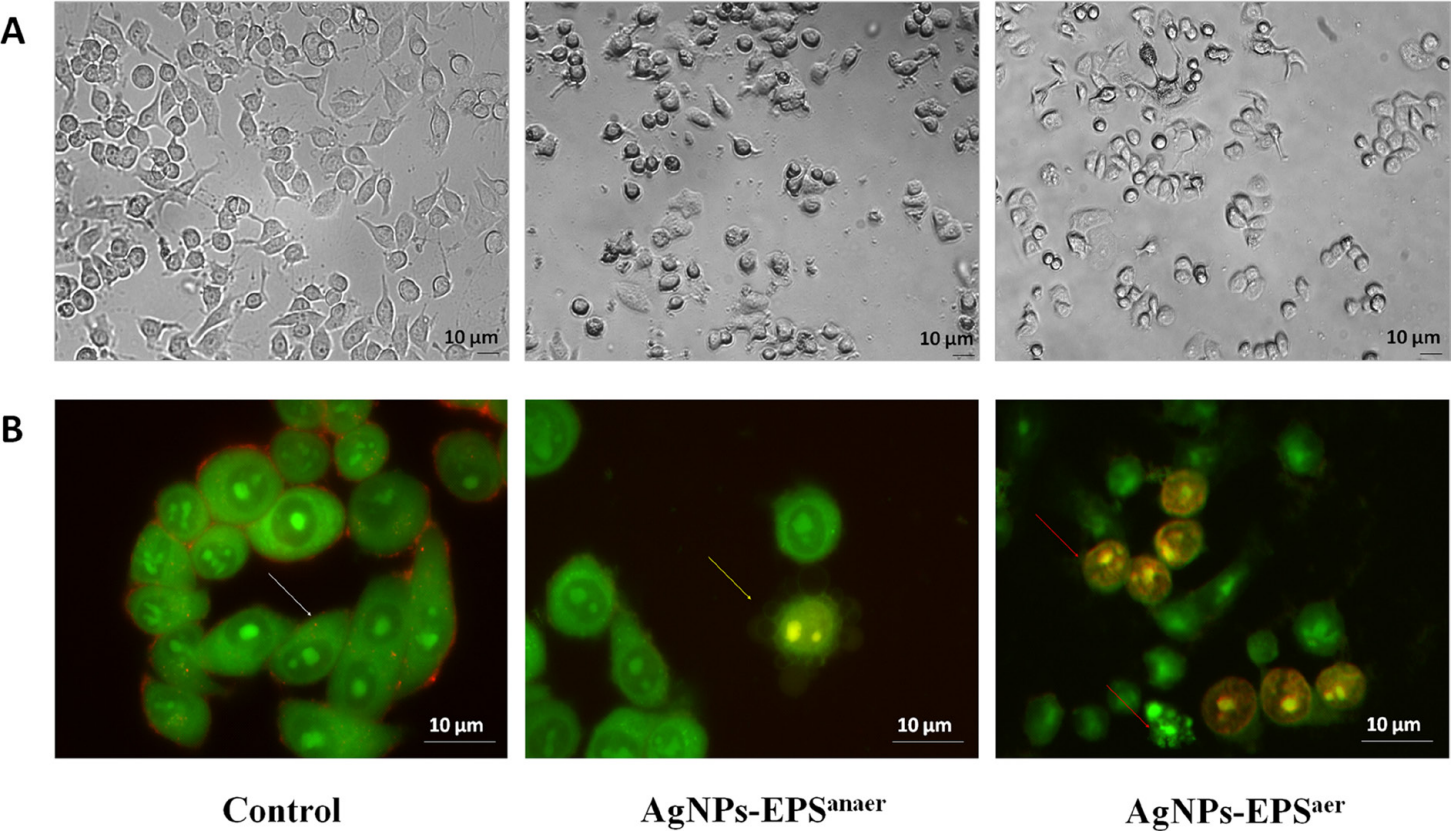
Morphological changes in SKBR3 cells treated with AgNPs-EPS (**A**) Inverted phase-contrast micrographs Magnification 200X. (**B**) Fluorescence microscope micrographs after AO/EB double staining. Magnification 680X. Viable cells (blank arrow), membranes blebbing (yellow arrow), loss of membrane integrity, condensed chromatin and nuclei stained with EB (red arrows). Each experiment was performed in triplicate (*n* = 3) and generated similar morphological features.

The morphology of SKBR3 cells treated with either AgNPs-EPS^aer^ and AgNPs-EPS^anaer^ were significantly changed in comparison to the untreated control cells: treated cells showed apoptotic-like symptoms such as cell shrinkage and detachment from the adjacent cells especially in the case of AgNPs-EPS^aer^ treatment. Moreover, irregularity in shape, cytoplasmic blebbing, intracellular vacuoles, cellular debris and nuclear chromatin condensation were clearly observed (Figure 3A). No morphological changes were observed for free-metal EPS (data not shown). AO/EB staining technique was used to verify whether the AgNPs-EPS-cell death is due to apoptotic induction or unspecific necrosis. As expected (Figure 3B), the control cells emitted only green fluorescence due to the AO staining. On the contrary, the AgNPs-EPS treated cells exhibited orange fluorescence (sensitive to pH lowering) due to the loss in membrane integrity. In particular, in AgNPs-EPS^anaer^ treated cells, only membrane blebbing and condensed chromatin were observed, while in AgNPs-EPS^aer^ treated cells, loss of membrane integrity, condensed chromatin and nuclei stained with EB, were observed.

### Effect of AgNPs-EPS treatment on intracellular oxygen species

**Figure 4:**
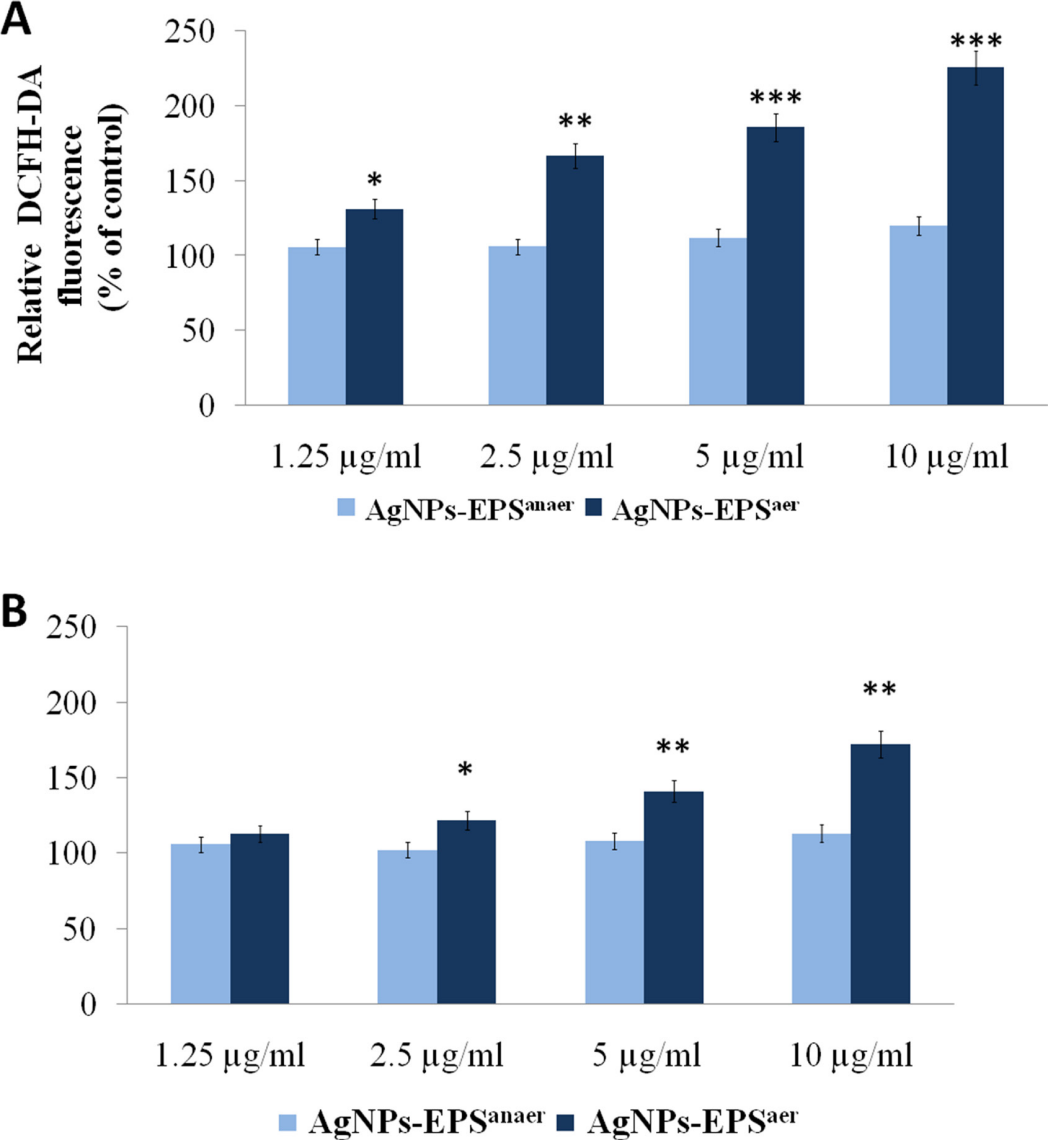
Effect of AgNPs-EPS treatment on intracellular ROS generation ROS were measured using DCFH-DA (**A**) and DHE (**B**) and quantified through spectrofluorometry. Data were normalized for cell number determined by MTT assay and expressed as percentage in respect to the untreated cells used as control. Statistical significance was assessed by the Student’s *t*-test: ^*^p < 0.05 was considered significant, ^**^p < 0.01 highly significant and ^***^p < 0.001 very highly significant. The data are expressed as mean value ± SD.

Elevation of intracellular ROS levels is known to induce oxidative stress and cell death [34, 35]. Several studied reported that AgNP cytotoxicity is primarily the result of oxidative stress [36, 37], which in turn induces cell death through several supposed molecular mechanisms such as activation of intrinsic pathway of apoptosis, autophagy or both [38–41]. We investigated the effect of AgNPs-EPS on intracellular levels of ROS by dichlorodihydrofluorescein diacetate (DCFH-DA) and Dihydroethidium (DHE) assays, two generic indicators of cellular ROS levels. As shown in Figure 4, after 24 h of exposure AgNPs-EPS^aer^ induced a remarkable ROS increase in a concentration-dependent manner. Compared to the control cells, a maximum of 2.25 and 1.75 fold increases in ROS generation was observed with 10 µg/ml concentration of AgNPs-EPS^aer^ treatment, in DCFH-DA and DHE assay respectively, suggesting an important role of ROS in cell death induction. AgNPs-EPS^anaer^ treatment did not affect significantly intracellular levels of ROS in comparison to the control cells.

### Detection of Apoptosis and autophagy

**Figure 5:**
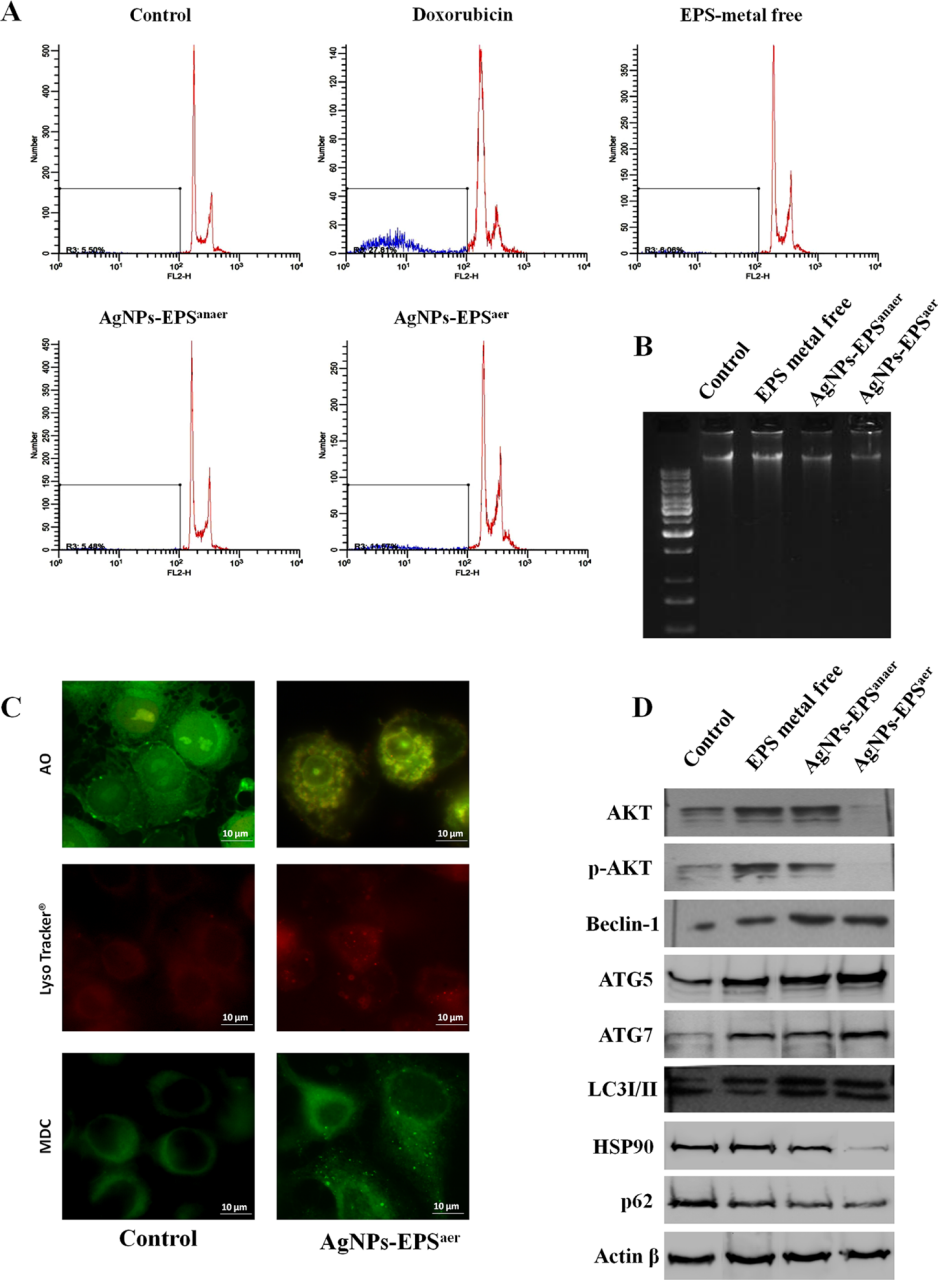
Detection of Apoptosis and autophagy on SKBR3 cells treated with AgNPs-EPS (**A**) Flow cytometry analysis of DNA distribution in propidium iodide-stained cells. (**B**) Genomic DNA separated on 1% agarose gels, and visualized by gel red staining. (**C**) Fluorescence micrographs after Acridine Orange, Monodancylcadaverine and Lysotracker staining for labeling of acidic vesicular organelles (AVO), autophagosomes/autolysosomes respectively. (**D**) Western blot analysis showing the effect of AgNPs-EPS treatment on the expression of autophagic protein markers. Actin was used as loading control.

We examined whether AgNPs-EPS-induced cell death occurs via apoptosis, autophagy, or both. Flow cytometry analysis (Figure 5A) revealed that only the 11.57% of AgNPs-EPS^aer^ treated cells underwent to apoptosis compared to the 5.50% in untreated cells and doxorubicin treated cells (27.81%), while no apoptosis was recorded for AgNPs-EPS^anaer^ treated cells (5.58%) and for free-metal EPS treated cells (6.06%). Accordingly, no DNA fragmentation was evident in SKBR3 cells after 24 h of Ag-NPs-EPS exposure (Figure 5B), suggesting that apoptosis was not prominent in AgNPs-EPS^aer^ -induced cell death, at least in the first 24 h of treatment. Therefore, three different staining specific for lysosomal/vacuolar activity include Lysotracker^®^ (a fluorescent acidotropic probe used for labeling acidic organelles in live cells such as lysosomes/vacuoles), MonoDansylCadaverine (MDC) (a weak base able to label autophagosomes and autolysosomes) and Acridine Orange (AO) (a fluorescent basic dye that accumulates in acidic compartments, such as lysosomes and vacuole) were used to investigate autophagy. AgNPs-EPS^aer^ treated cells (Figure 5C) showed autophagolysosomes in the cytoplasm detected by fluorescence microscopy as bright punctate dots [42, 43]. Moreover the expression of autophagic markers such as AKT, p-AKT, ATG5, ATG7, HSP90, P62, LC3I/II protein and beclin I, were analyzed by Western blot (Figure 5D). As expected, we observed a concomitant down regulation of AKT, p-AKT, p62 and HSP90 expression and an up-regulation of ATG5, ATG7, LC3-II and beclin-1 in AgNPs-EPS^aer^ treated cells, indicating a prominent mechanism of autophagic cell death.

### Effect of AgNPs-EPS on SKBR3 proteome

**Figure 6:**
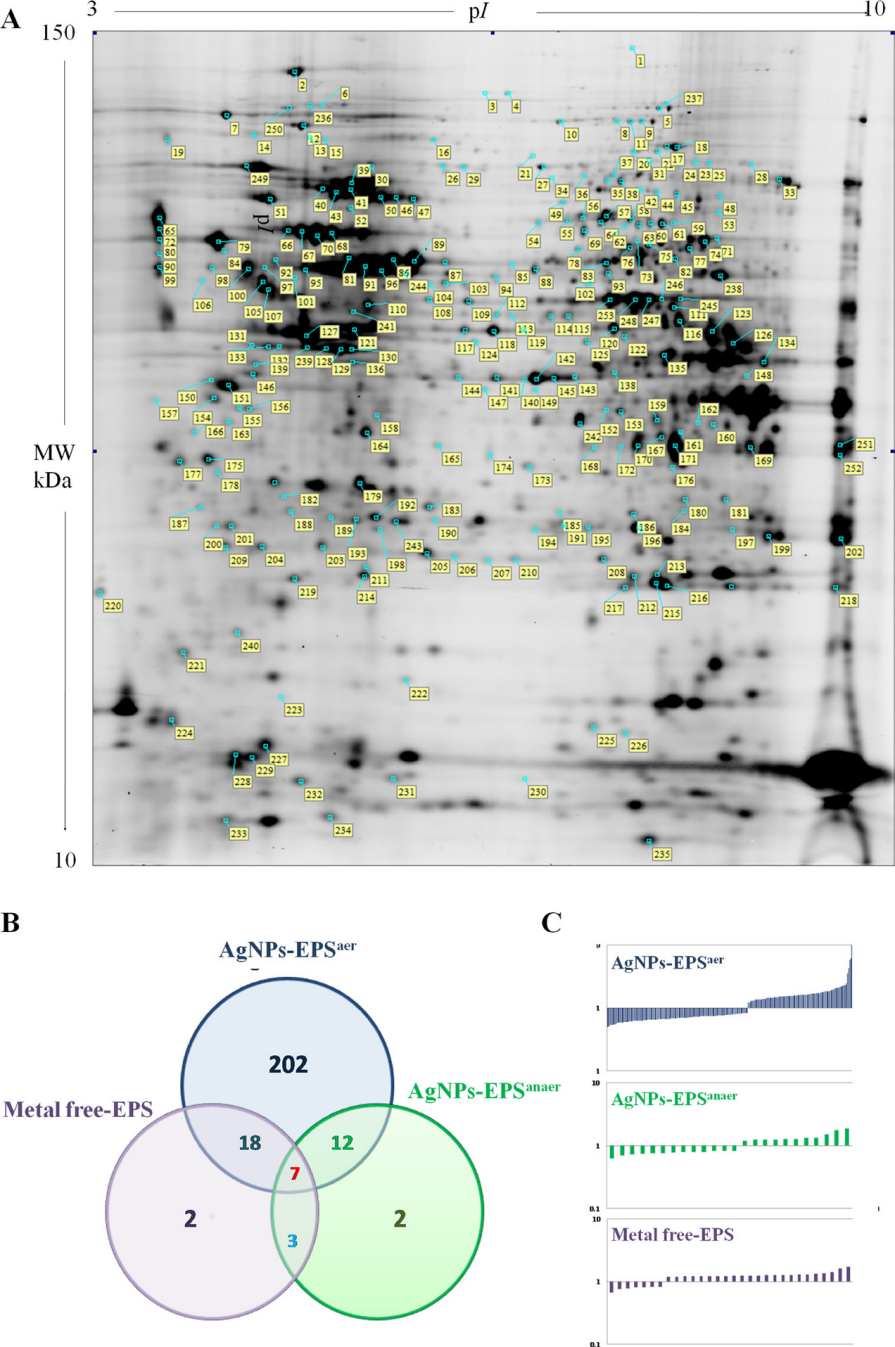
Differential proteomic analysis of SKBR3 cells treated with AgNPs-EPS (**A**) Proteomic map of SKBR3 control cells. Labels indicate the 253 differential protein spots. (**B**) Eulero Venn diagram showing the differentially expressed proteins selected on the threshold setting in the studied groups; (**C**) Histograms showing the up and down-regulated proteins plotted as fold change compared to untreated cells.

**Figure 7:**
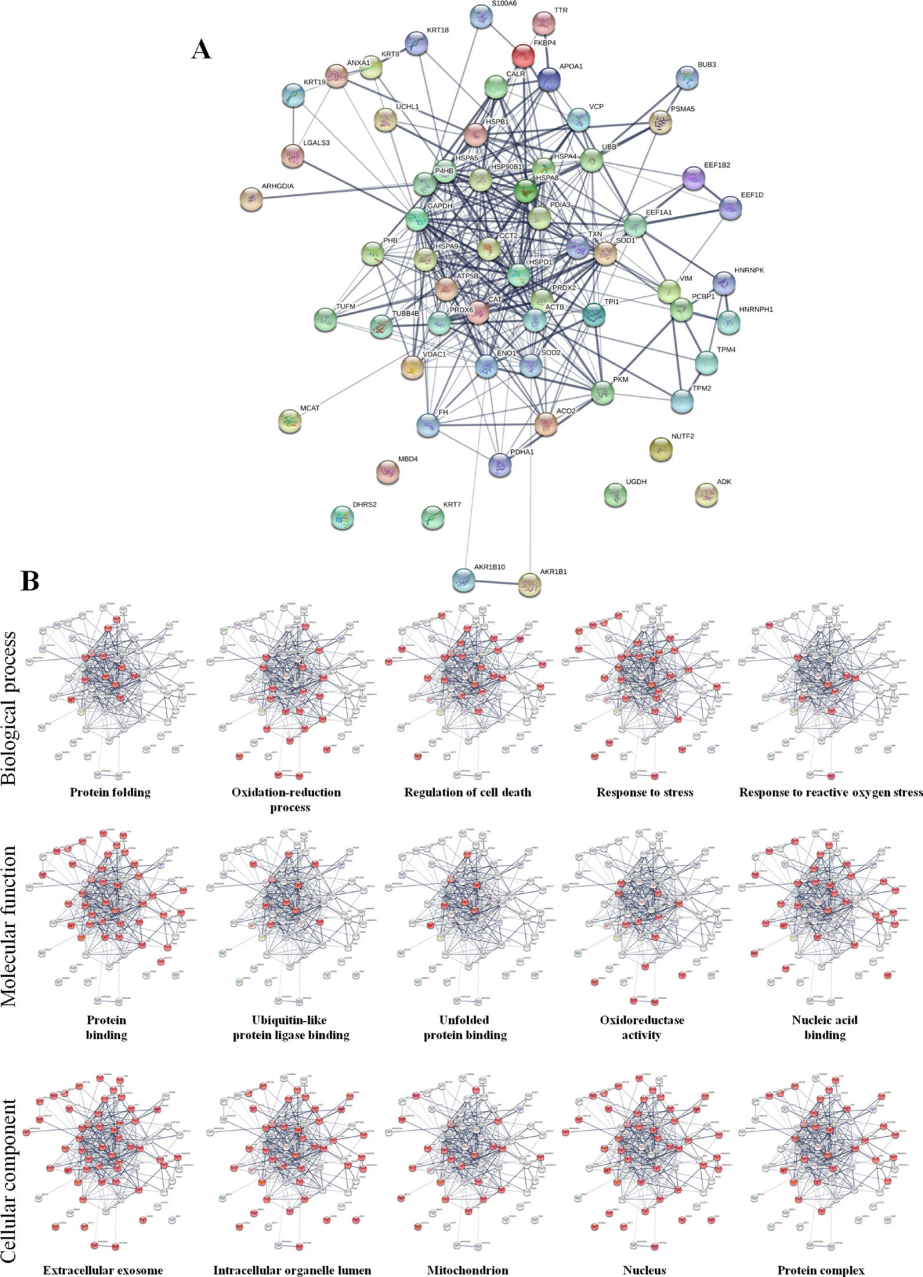
Protein-protein interaction networks (**A**) Functional interaction network of AgNPs-EPS^aer^ regulated proteins created by String algorithm. Stronger interactions are represented by thicker lines. (**B**) Miniatures of functional network sorted by enriched Gene Ontology terms: biological processes, molecular functions and cellular components. For each group the involved proteins are highlighted in red.

**Figure 8:**
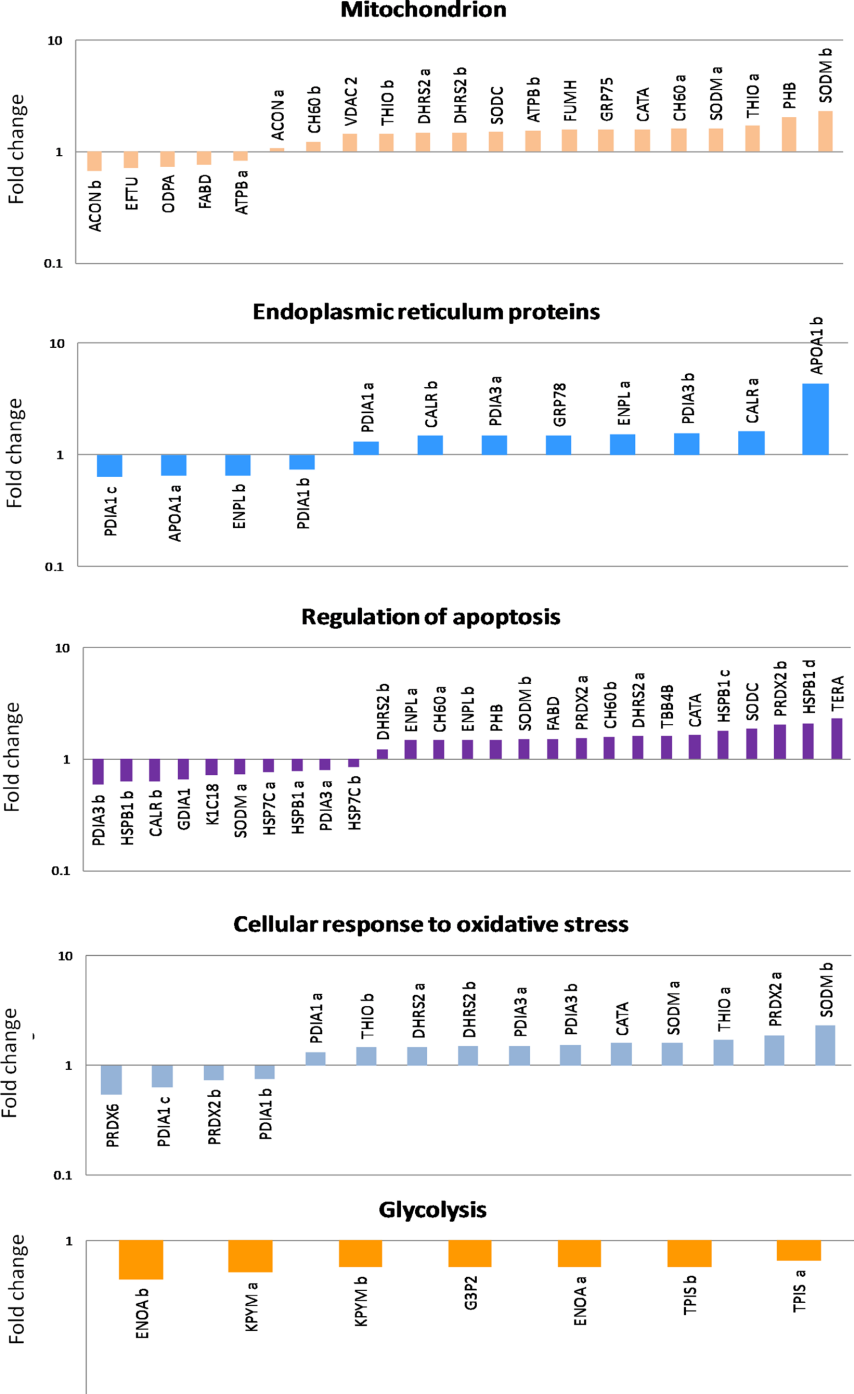
Histograms of the differentially identified proteins in SKBR3 AgNPs-EPS^aer^ treated cells, sorted into functional classes and plotted as fold change compared to untreated cells

Differential proteomic analysis (2D-DIGE) was performed to detect protein modulations induced by both AgNPs-EPS on SKBR-3 cells, after 24 h of treatments in order to unveiling molecular pathways involved in AgNPs-EPS^aer^ cytotoxicity. A total of six gels were run to achieve a statistically significant measure of the differences in protein expression between the analyzed groups. Protein spots were quantified, normalized and inter-gel matched and only differentially expressed proteins with the threshold settings with at least fold change ≥ 1.3 and *p*-value < 0.01, were considered regulated (Supplementary Table 1). Figure 6A shows a prototype of proteomic map of SKBR3 cells with 253 labels corresponding to the protein spots showed a significant difference. Among the differentially expressed spots (Figure 6B), 202, corresponding to 80%, were selectively modulated in AgNPs-EPS^aer^ treated cells, only two proteins spots were modulated selectively in AgNPs-EPS^anaer^ and only two in EPS free metal treated cells, confirming the prominent biological activity of AgNPs-EPS^aer^. Overlapping modulations are shown, also. The trend of up and down regulated proteins compared to untreated cells are shown in Figure 6C. Protein identification was performed by MALDI TOF-MS/MS analysis. We successfully identified 118 protein spot, corresponding to 83 proteins (Supplementary Table 2). Several proteins were found in different isoforms and then detected in multiple spots. In order to construct protein-protein interaction networks regulated by AgNPs-EPS^aer^ we used STRING database [44]. Figure 7A depict the interaction networks of the AgNPs-EPS^aer^ regulated protein, in which is possible to observe a group of proteins biologically connected, with several interactions among themselves. Functional enrichments analysis performed by using gene ontology terms, allow us to better understand the molecular mechanisms underlying the cellular responses triggered by AgNPs-EPS^aer^: Figure 7B shows the protein clusters involved in different biological processes (protein folding, oxido-reduction processes, regulation of cell death, response to stress and in particular to reactive oxygen stress), molecular functions (essentially binding capability e.g proteins and nucleic acid, and oxidoreductase activity) and cellular component (extracellular exosomes, intracellular organelles lumen, mitochondrion, nucleus). Moreover, the biological meaning and functional pathways were extracted by using DAVID Bioinformatics Resources [45], where a high number of proteins with specific biochemical functions were found. In particular, the majority of the identified proteins can be classified into 5 different groups namely: mitochondrial proteins, endoplasmic reticulum lumen proteins, proteins involved in regulation of apoptosis, proteins involved in the response to oxidative stress and glycolytic proteins. Figure 8 reports the histograms of protein level variations following the AgNPs-EPS^aer^ treatment, compared with the untreated cells. Interestingly, proteomics results highlights important pathways involved in the mechanism of action of AgNPs-EPS^aer^ on SKBR3 cells and strengthens our previous observations on its biological function. In particular the differentially proteins identified can efficiently explain the cellular effects of AgNPs-EPS^aer^. Infact accumulating evidence suggests that protein folding and generation of reactive oxygen species (ROS) as a byproduct of protein oxidation in the ER are closely linked events [46]. Persistent oxidative stress and protein misfolding initiate cell death cascades, involving mitochondrial disfunctions; moreover, mitochondrial turnover is dependent on autophagy. Collectively, the dow-regulation of glycolitic enzymes suggest a partial reversion of the so called Warburg effect, first described by Otto Warburg, and considered an important cancer hallmark [47].

### Uptake of AgNPs-EPSaer by SKBR3 cells

**Figure 9:**
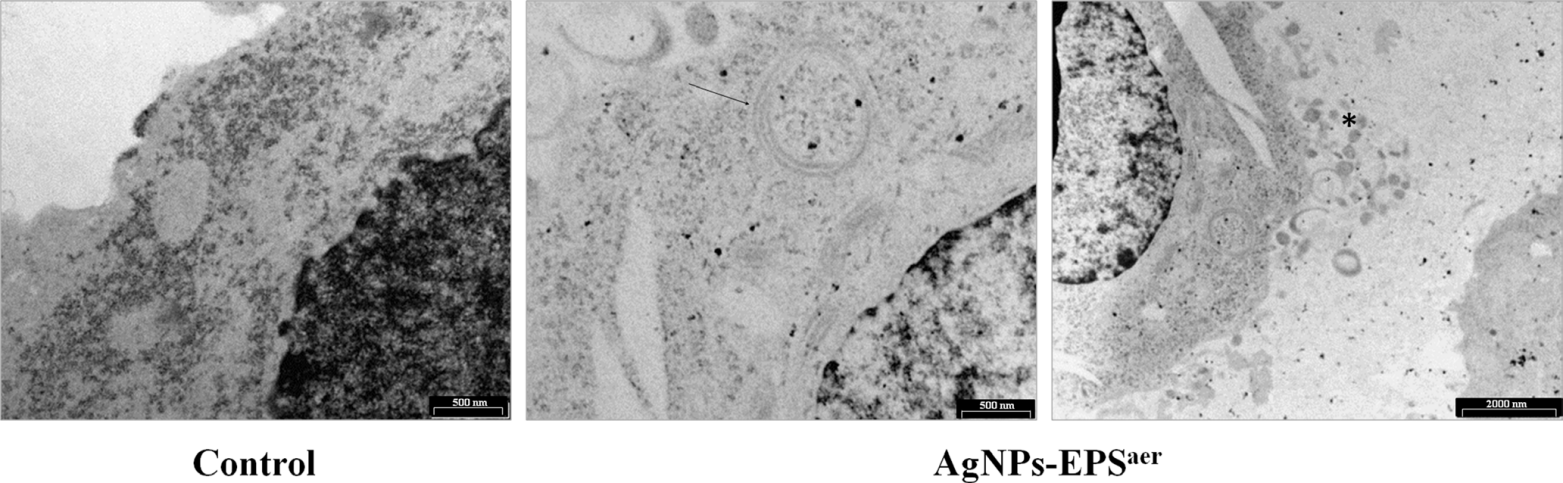
TEM micrographs of SKBR3 cells treated with AgNPs-EPS^aer^ Arrow indicates endosomes while asterisk indicates vesicles and pseudopodia.

TEM micrographs (Figure 9) showed that AgNPs-EPS^aer^ treated cells exhibited numerous endosomes with engulfed nanoparticles and autophagic vacuoles filled with structures resembling mitochondria. Moreover numerous exocytic vesicles and pseudopodia were also observed. Only few nanoparticles were observed within the nucleus, because the matrix is more electron dense than the cytoplasm and it is more difficult to distinguish the single silver nanoparticles.

### Determination of total silver in cell fractions

**Figure 10:**
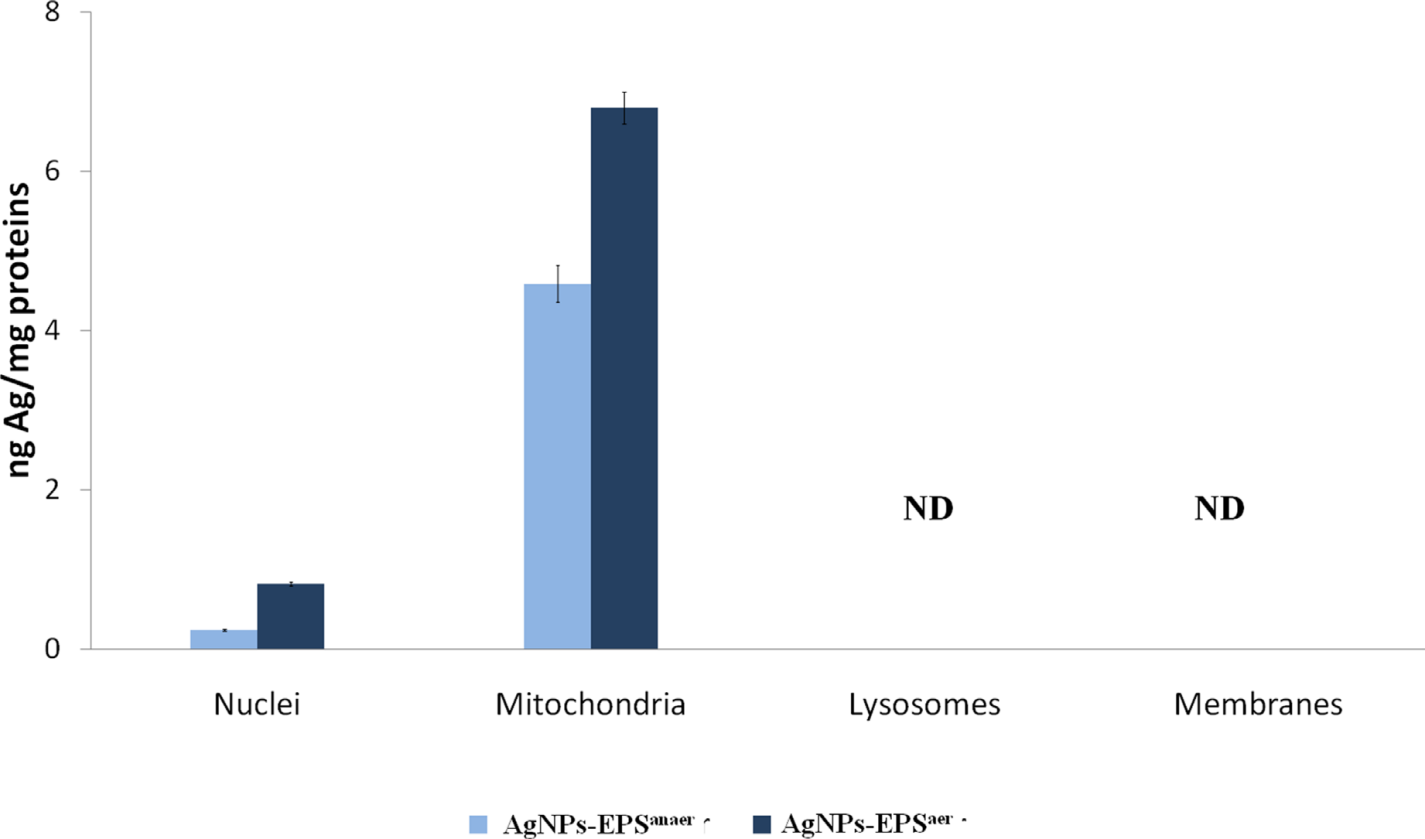
Determination of total silver by ICP in cell fractions Data were normalized for protein concentration. ND means not detected.

Inductively coupled plasma atomic emission spectrometry (ICP), was used to measure the concentration of total silver in cell fractions (nuclei, mitochondria, lysosomes and membranes) obtained by sequential centrifugation of AgNPs treated cells for 24 h. As expected, higher Ag concentration was detected in AgNPs-EPS^aer^ treated cells compared to AgNPs-EPS^anaer^ treated cells. Interestingly, tot-Ag was detected only in mitochondria and nuclei fractions, with a higher concentration in mitochondria with respect to nuclei (Figure 10), suggesting a prominent role of mitochondria in AgNPs-EPS^aer^-toxicity. Mitochondria, resembling to bacteria, likely have a similar mechanism of transportation for Ag^+^ in *E.coli* [24].

### Excitation emission spectroscopy of DNA

**Figure 11:**
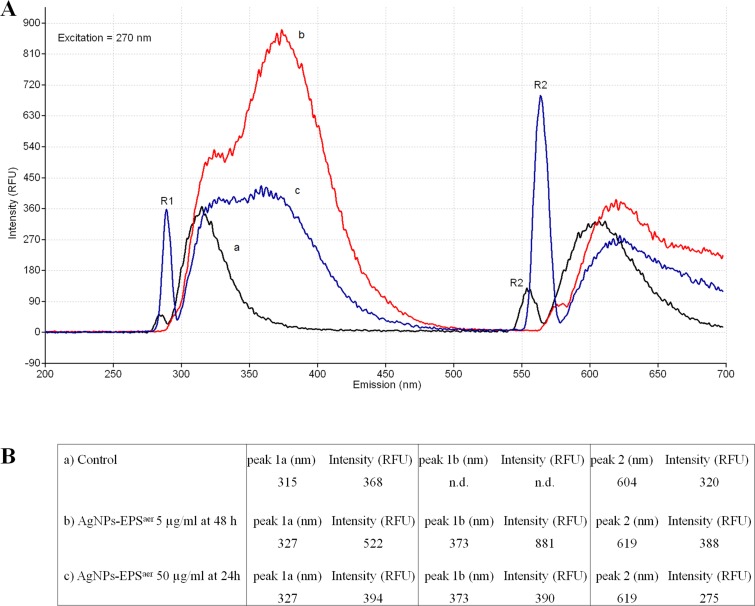
Excitation-emission spectroscopy of DNA Determination of intrinsic DNA fluorescence. The DNA was extracted from SKBR3 treated and untreated with AgNPs-EPsaer. The Eex was set at 270 nm and the Eem were ranging from 200 to 700 nm. The relative fluorescence units (RFU) of intensities and the 3 emission peaks (1a, 1b and 2) were reported. R1 and R2 corresponded to the Rayleigh scattering peaks.

The interaction of Ag^+^ with DNA was studied with EEM spectroscopy. For this purpose the three dimensional excitation emission matrix (EEM) fluorescence spectroscopy was used to characterise and distinguish the DNA from the Ag-DNA. This fluorescence method is specific, sensitive and reliable enough to characterise and distinguish even similar synthetic organic dyes [48]. The fingerprints of EEM spectra of DNA extracted from cell line SKBR3 show significant changes in the E_ex_/E_em_ contour for exposed and unexposed cells. The intrinsic fluorescence of DNA from untreated cells which are excited with E_ex_ = 270 nm produces two sharp E_em_ peaks: the first peak centred at 315 nm and the second peak centred at 604 nm (Figure 11). The same pattern was detected in exposed cells to IC_50_ of AgNPs-EPS^aer^ for 24 h (data not shown). Accordingly to ICP results, these results reinforce the idea that at 24h of treatment DNA is not the primary target of AgNPs-EPs^aer^ treatment. The EEM contours have significantly changed when the cell remains exposed to AgNPs-EPs^aer^ with an higher concentration (50 mg/ml) or for a longer time (48 h). In these conditions the first peak doubled in two peaks namely (1a) and (1b), with an important shift of peak (1a) from 315 to 327 nm and with a new peak (1b) at 373 nm. The second DNA peak was shifted from 604 nm of the control cells to 619 nm of silver exposed cells. The intensity (RFU) of peaks, the shifts, and in particular the doubling of first peak are to be considered as caused by DNA interaction with Ag+. These peaks are similar to those produced *in vivo* with *E.coli* [24] and *in vitro* by Ag^+^ complexed by synthetic DNA oligonucleotides [49, 50].These results could indicate that in the long-time treatment with AgNPs-EPs^aer^ increase and/or stabilise the interaction of silver with DNA inducing cell death [51].

## DISCUSSION

Metal nanoparticles are versatile agents with a variety of applications and in particular in nanomedicine for highly sensitive diagnostic assays, thermal ablation, radiotherapy enhancement including drug and gene delivery [14]. In this study, two biogenerated AgNPs-EPS has been used to induce toxicity and to study the mechanisms of toxicity in a breast cancer cell line SKBR3. Both AgNPs-EPS were already characterized and used for antimicrobial activities [24]. The most important characteristics of silver nanoparticles used in this study are: the spherical shape with an average size of 11 ± 5 nm. The concentration of total silver in AgNPs-EPS^aer^ is 12.1 % while in AgNPs-EPS^anaer^ is 5.2 %, whereas the relative productions of AgNPs-EPS^aer^ and AgNPs-EPS^anaer^ were 170 and 312 mg/L, respectively. Information on reactivity of AgNPs and the release of Ag(I) species at the AgNPs-EPS/water interface was obtained by combining SECM and voltammetric experiments [25]. It is interesting to note that the amount of Ag(I) released from AgNPs-EPS^aer^ is sensibly higher (4–6 times) than that of AgNPs-EPS^anaer^. The composition of the AgNPs-EPS materials are differently rich of Ag(I)-NPs or Ag(0)-NPs. As expected, Ag(I) forms predominate in the AgNPs-EPS^aer^ material, conceivable due to the presence of oxygen, which could promote the oxidation of Ag(0)NPs to provide easier diffusible Ag(I) species. Accordingly to their chemical and physical properties, AgNPs-EPS^aer^ showed most important biological activity in term of cytotoxic, morphologic and proteomic effects. We also verified a selective action of AgNPs-EPS^aer^ in SKBR3 breast cancer cells compared to the non tumoral mammary cell line HB2. Since cancer cells display an altered metabolism and increased glucose uptake, irrespective of oxygen availability (aerobic glycolysis or Warburg effect) [52], we believe that they are able to detect the sugars present in the EPS and to pick it with greater efficiency compared to normal cells. Recently, carbohydrates have been considered as biomimetic functional molecules on the surface of nanoparticles [53] also because they trigger cellular uptake via specific receptors or endocytosis. TEM analysis showed that AgNPs-EPS^aer^ uptake occurs through endocytosis and, as suggested by colony formation assay, the effects are evident after 1h of treatment. Moreover, AgNPs-EPS^aer^ treatment inhibited migration and invasion capability of tumoral cells, considered as a cancer hallmark, suggesting a potential anticancer effect.

Findings from several studies reported that both the AgNPs and Ag^+^ released by AgNPs are involved in the mechanism of cytotoxicity in different ways: infact, AgNPs probably provide a perfect surface outside the mitochondria for the univalent reduction of oxygen to superoxide from electron through the electron transport chain. On the other hand, Ag^+^ binds to proteins and DNA, interfering with their functions [54]. Moreover, ROS generation and oxidative stress occur as an early event leading to NP-induced toxicity [38].

Our results point in the same direction suggesting that the cellular response to AgNPs-EPS^aer^ exposure is the sum of events triggered by a direct effect of the cellular uptake of AgNPs, as verified by TEM analysis, induction of intracellular ROS and an indirect effect of free Ag^+^ ions released mainly in mitochondria and secondarily in the nuclei, where they interact with DNA. To the best of our knowledge this is the first study reporting quali-quantitative evidences about the effects of siver ions released by nanoparticles in different cell compartments.

It is generally accepted that ROS induce autophagy and/or apoptosis. Moreover, it has been documented that autophagy may act as enabler of apoptosis, contributing in certain morphological and cellular events that take place in apoptotic cell death [55]. Autophagic cell death has morphologic and biochemical features distinguishing it from both apoptosis and necrosis. Autophagy consists of several sequential steps starting with the autophagosome formation and then progresses to autophagolysosomes through the fusion of acidic lysosomes with autophagosomes [56–58]. Several proteins can be used as markers to study autophagic flux: in particular, during autophagy, ATG proteins (eg ATG5 and ATG7) and Beclin -1, have an essential role in the autophagosome formation [59]. Later, autophagosomes (LC3-containing vacuoles) fuse with lysosomes to cause the degradation of their contents. The protein p62/SQSTM1 binds directly to LC3 and is itself degraded [60, 61]. Moreover, inhibition of HSP90 plays an important role in autophagy inducing degradation of Hsp90 client proteins, such as AKT [62, 63]. PI3K/AKT/mTOR axis downregulation lead to the activation of autophagy and the inhibition of cell proliferation, also [63]. We found that SKBR3 treated with AgNPs-EPS^aer^ underwent morphologic and biochemical changes consistent with the induction of autophagy and secondarily of apoptosis, as suggested by the upregulation of lysosomes, autophagolysosomes and acidic vescicular organelles, the down regulation of AKT, p-AKT, HSP90 and p62 the increment of ATG5, ATG7 and beclin-1 and the conversion of LC3-I to LC3-II. Concerning the apoptosis, only the 11% of treated cells underwent to apoptotic cell death and accordingly, no DNA fragmentation was evident in our analysis at 24h, although the increment of Ag^+^-DNA binding was evident after 48h of treatment.

Several evidences suggested that activation of autophagy, is also dependent by the accumulation of oxidatively damaged proteins and subsequent endoplasmic reticulum stress, or mitochondrial damage [34, 64–66]. Our proteomic results are in agreement with these observations. The differentially proteins identified so far, highlights important pathways involved in the mechanism of action of AgNPs-EPS^aer^. The endoplasmic reticulum (ER) is a well-orchestrated protein-folding machine composed of protein chaperones, proteins that catalyze protein folding, and sensors that detect the presence of misfolded or unfolded proteins. The protein folding and generation of reactive oxygen species (ROS) as a byproduct of protein oxidation in the ER are closely linked events, and persistent oxidative stress and protein misfolding initiate cell death. Autophagy is emerging as an important mediator of pathological responses and engages in cross-talk with ROS. Oxidative stress is inseparably linked to mitochondrial dysfunction, as mitochondria are both generators of and targets for reactive species. Moreover, the mitochondrial turnover is dependent on autophagy. We believe that proteomic pathways disclosed in this study represent an important advance about the mechanisms of AgNPs-EPS^aer^ induced toxicity according to a modern omic-research direction in cancer therapy.

In conclusion, our results suggest a so-called “Trojan-horse” mechanism [67], in which AgNPs-EPS^aer^ internalized within the cells access the mitochondria and nuclei where release Ag ions, which determine oxidative stress and cell death. The crosstalk between autophagy, redox signaling and mitochondrial dysfunction is not well understood and further efforts are necessary to analyze the oxidative stress-ER stress-mitochondria connectivity and apoptosis/autophagy. These interesting results encourage further studies to highlight the *in vivo* effects on breast cancer cells.

## MATERIALS AND METHODS

### Culture conditions production of two AgNPs-EPS

Cells of *K. oxytoca* DSM 29614 strain were stored in cryovials at –80°C in 25% glycerol solution until they were revitalized in Nutrient Broth (Difco), When the strain was grown under anaerobic and aerobic conditions the NAC medium, buffered at pH 7.8, was used (2.5 g/L NaHCO_3_, 1.5 g/L ammonium acetate, 1.5 g/L MgSO_4_.7H_2_O, 0.6 g/L NaH_2_PO_4_, 0.1 g/L potassium acetate, and 14.7 g.l^-1^ sodium citrate) [22]*.* The anaerobic medium was cooled under N_2_ flux and then was sealed. An overnight pre inoculum of cells was prepared and inoculated both into this oxygen-free medium and that aerated medium with 10 ml of DSM 29614 culture (OD_600nm_ = 1.0) and incubated at 30°C. After 3 days incubation for aerobic medium and after 6 days for anaerobic medium, 50 mg of Ag^+^, such as AgNO_3_, was added in both cultures. The growth of the strain was followed by determining total proteins according to Bradford micro-method recommended by Bio-Rad reagent. After 2 days from Ag^+^ additions both precipitates were recovered by a gently centrifugation (1000 *g* x 20 min). The precipitated cells were removed by further centrifugations. The polysaccharide fraction was washed twice with milliQ water and the polysaccharide was precipitated with 70% ethanol solution overnight at 4°C. Both ethanol precipitates were dried out under vacuum and grinded in a mortar. Thereafter the two biogenerated silver nanoparticles were designed as AgNPs-EPS^aer^ and of AgNPs-EPS^anaer^.

### Cell culture and treatments

SKBR3, 8701-BC, HT29 and HCT116, were cultured in RPMI 1640 media (Gibco, Paisley, UK), while CaCo2 cells were cultured in DMEM medium, supplemented with 10% heat-inactivated fetal bovine serum and 100U/mL penicillin and 100 µg/mL streptomycin, as already described [33, 68–71]. The human mammary epithelial cell line HB2, already characterized for proteomic profile [70], was grown in Dulbecco’s modified Eagle’s medium (DMEM) supplemented with 10 % fetal bovine serum, 100 U/mL penicillin and 100 µg/mL streptomycin, hydrocortisone (5 μg/mL) and insulin (10 μg/mL). All culture cells were maintained at 37°C and 5% CO_2_. Cells were seeded at cell density of 2 × 10^4^/cm^2^, and to 70% confluence they were treated with different concentrations of AgNPs-EPS^anaer^, AgNPs-EPS^aer^ and metal-free EPS (produced in absence of metal), for 24 h for all experiments. Where not expressed, the cells were treated with the corresponding IC_50_ values.

### Cell viability assay

The cytotoxic activity of both NPs-EPS was determined using MTT assay (Promega, Madison, WI, USA), as previously described [69, 72]. Briefly, cells were plated at 5 × 10^3^ cells/well in 96-well plates. NPs-EPS, diluted to the desiderated concentrations in culture medium, were added to the wells with respective vehicle control (H_2_O). Doxorubicin hydrochloride (Sigma, st. Louis, MO) was used as reference drug. After 24 h of incubation 20 µL of the Cell titer 96^®^AQ_ueous_ reagent was added to each well after three washes with phosphate buffer saline (PBS) and incubated for 1-4 h at 37 °C in a CO_2_ incubator. The absorbance was recorded at 490 nm using a 96-well plate reader (Spark^®^ 20M, Tecan Trading AG, Switzerland). The percentage of cell viability was calculated with respect to untreated control cells for each treatment after subtraction of the blank. The concentration necessary for 50% of growth inhibition (IC_50_) was calculated using a dose–response model, which was obtained from sigmoidal fitting of response curves of percent inhibition versus logarithmic concentration of DOSs using Graph Pad Prism software. Each result was the mean value of three different experiments performed in triplicate.

### Colony formation assay

This assay identifies the cell populations that survive following a cytotoxic treatment. Cells were seeded at a density of 200, 400 and 800 cells in 6-well plates. After 2 h, the cells were treated for 1 h and for 24 h with 5 and 50 μg/L of AgNPs-EPS. At the end of the incubation period, medium was replaced with fresh medium and cells were incubated for 10 days. Colonies were fixed and stained with a mixture of 6.0% glutaraldehyde and 0.5% crystal violet and then counted microscopically using 10× high power fields. Clonogenic index was then calculated as the ratio of plating efficiency of treated cells on wells divided by the number of cells in the control wells.

### Cell migration

Cell migration was studied by using an *in vitro* scratch assay. SKBR3 cells were seeded on 24-well tissue culture plates and grown to 100% confluence. Wounds were created by scraping the monolayer of cells with a sterile pipette tip, washed with PBS to remove the floating cells and incubated with fresh medium in the presence or absence of AgNPs-EPS. The images of scratched area were captured (at 40× magnification) using an inverted microscope equipped with digital camera immediately after wounding and after 6 and 24 h. The scratched area was calculated by Image J software.

### Gelatin zymography

SKBR3 cells (5 × 10^4^ cells/well) were seeded on 6-well plates, grown to 90% of confluence and treated for 24h with AgNPs-EPS IC_50_ in a serum-free medium. Conditioned media were collected, centrifuged to remove cellular debris, dialyzed and lyophilized. Equal amounts of total proteins (5 µg) were separated by electrophoresis, on a 7.5% sodium dodecyl sulfate (SDS)–polyacrylamide gel containing 0.1% gelatin, under non-reducing conditions. After electrophoresis, the gels were washed for 1h with a buffer containing 50 mM Tris-HCl, pH 7.5 and 2.5% Triton X-100, to remove the SDS and then incubated with activation buffer containing 50 mM Tris-HCl, pH 7.5, 150 mM NaCl, 5 mM CaCl_2_, for 18h at 37 °C. Gel was stained with Coomassie Brilliant Blue G 250 and de-stained with H_2_O milliQ. The bands intensity were measured with Image J software.

### Morphological assessment and Acridine orange/Ethidium bromide (AO/EB)

SKBR3 cells were seeded in 12 well culture plates with a cell density of 1 × 10^5^ cells/well. After 24 h cells were incubated for 24 h with the IC_50_ concentration of the AgNPs-EPS^aer^, and observed for morphological changes assessment under a phase contrast inverted microscope (*Carl Zeiss*) at 400X magnification. For AO/EB, the cells were seeded at the same conditions in a cover slip and after treatments, cells were washed twice with PBS and stained for few minutes with 200 μL of the Acridine Orange (100μg/mL), Ethidium Bromide (100μg/mL) mixture (1:1, v/v). Cells were immediately observed under a fluorescent microscopy (Carl Zeiss) at 630X magnification.

### Evaluation of ROS generation

Intracellular ROS levels were measured by using 2′,7′-dichlorodihydrofluorescein diacetate (DCFH-DA) and Dihydroethidium (DHE) assays. DCFH-DA is routinely used to measure intracellular generation of H_2_O_2_ and other oxidants; it is a cell-permeable probe, hydrolyzed intracellularly to the DCFH. Two-electron oxidation of DCFH results in the formation of a fluorescent product, dichlorofluorescein (DCF). DHE is another widely used probe for detecting intracellular O_2_^-^.The red fluorescence formed from the two-electron oxidation product, is usually equated to intracellular superoxide formation. SKBR3 cells were plated in 96 well plates at a density of 5 × 10^3^/well, allowed to growth overnight and incubated for 24 h with different AgNPs-EPSs concentrations. At the end the medium was replaced with the culture medium containing DCFH-DA (10 *μ*M) and DHE (10 *μ*M) and incubated for 30 min at 37°C. Then the medium was replaced with PBS and the fluorescence intensity was analyzed by spectrofluorimeter with an excitation of 488 nm and emission wavelength of 525 nm for DCFH-DA and an excitation of 540 nm and emission wavelength of 590 nm for DHE. Data normalization was performed with parallel MTT assay.

### DNA extraction and flow cytomety

DNA was extracted from SKBR3 treated cells as previously described [73], with some modifications. The cells were detached from the flasks with trypsin and suspended in 0.5 ml of TE buffer (100 mM Tris-HCl, 100 mM EDTA pH 8). 50 µl of 10% SDS and 25 µl of 1 mg/ml Proteinase K were added to the cell suspension and incubated for 30 minutes at 55°C. Nucleic acids were extracted using phenol-chloroform, precipitated with 1/10V 3M Na-acetate and 2V absolute ethanol and resuspended in 100 µl TE buffer. The same quantity of extracted DNA was loaded on a 1% agarose gel prepared in 0.5X TE buffer containing gel red. Gel was visualized under UV light using a Bio-Rad Trans illuminator and photographed by using a Polaroid camera.

Apoptosis of SKBR3 cells after treatment with IC_50_ of AgNPs-EPS for 24 h was analyzed by flow cytometry essentially as described by Riccardi [74]. Briefly cells were fixed in 70% ethanol, washed in PBS and resuspended in DNA extraction buffer (200 mM Na2HPO4, 0.1% Triton X-100). After staining with Propidium Iodide (1µg/mL) for 30 min, fluorescence intensity was acquired in the FL2 channel by flow cytometry on a FACSCalibur flow cytometer (BD, New Jersey, USA). Data acquisition was performed with CellQuest Pro (BD) software, and analyzed with WinList software (Verity Software House, Topsham, USA).

### Fluorescence staining with Acridine Orange (AO), monodansylcadaverine (MDC) and Lysotracker^®^

SKBR3 cells were grown on coverslips and treated for 24 h with the IC_50_ concentration of AgNPs-EPS^aer^. Acidic vesicular organelles (AVOs) were labeled with 1 µg/ml of Acridine Orange, autophagosomes and autolysosomes with 0.05 mM of MDC (Sigma, St. Louis, MO, USA) while acidic compartments were labeled by incubating the cells with 75 nM of LysoTracker^®^ (Molecular Probes, Life Technologies) in the culture media for 1min at RT. After incubation, cells were washed several times with PBS and immediately analyzed by fluorescence microscopy using the appropriate filter system.

### Western blotting analysis

SKBR3 cells were treated for 24h with IC_50_ concentrations of AgNPs-EPS and with 200 µg/ml of free-metal EPS. After washing with PBS, cells were carefully scraped and incubated on ice for 30 min Lysis buffer containing 7 M urea, 2 mM Thiourea, 0.4 % w/v CHAPS, 1 % w/v 1,4-dithioerythritol (DTE) and 30mM TRIZMA base, pH 8.5. The total cellular lysate was centrifuged at 14,000 rpm for 10 min to clear cell debris and protein concentration determined by Bradford assay, as already reported [75, 76]. Protein samples (20 *μ*g/lane) were subjected to SDS polyacrylamide gel electrophoresis, then transferred to a nitrocellulose membrane (HyBond ECL, Amersham) and stained with Ponceau S (Sigma Aldrich). Western blotting analyses were performed using a rabbit polyclonal antibodies for Akt 1-2-3 and pAkt 1-2-3, a goat polyclonal antibody for Beclin 1, a mouse monoclonal antibody for Actin β, HSP90, MMP2 and MMP9 by Santa Cruz Biotechnology (Santa Cruz, CA, USA), a rabbit polyclonal antibody for LC3 by Sigma Aldrich and a rabbit polyclonal antibodies for p62, ATG5 and ATG7 by Cell Signaling Technology. Following incubation with the appropriate peroxidase-linked antibody, the reaction was revealed by the ECL detection system, using high performance films (Hyperfilm ECL, Amersham), as already described [68, 77]. The correct protein loading was ascertained by red Ponceau staining and immunoblotting for Actin *β*.

### Subcellular separation for Ag+ release

After treatments, SKBR3 cells were detached from the flasks with a scaper and resuspended in Sucrose buffer (5mM Tris-HCl pH 7.4, 0.32M Sucrose and 0.001 mg/mL Protease/phosphatase inhibitor). Nuclei were pelleted by centrifugation at 1000 g for 20 minutes; mitochondria from the post-nuclear supernatants were recovered by centrifugation at 8500 g for 30 minutes; lysosome by centrifugation at 20000 g for 30 minutes and membranes by centrifugation at 105000 g for 90 minutes. The Ag^+^ release from subcellular components was measured by scanning electrochemical microscopy and anodic stripping voltammetry (ASV) using a CHI920B workstation (CH Instruments), as already described [24, 25].

### Determination of total Ag

Total silver was determined in 1 mg sample of pulverized AgNPs-EPS^aer^, AgNPs-EPS^anaer^ and in subcellular fractions of breast cancer cell line SKBR3. Samples were digested with 2 mL of aqua regia, heating at 60° until a solution was obtained*.* Total Ag was determined by Inductively Coupled Plasma Atomic Emission Spectrometry, ICP-AES (Optima 3100, Perkin Elmer) [78].

### 2D-DIGE analysis

Protein samples were labeled for 2D-DIGE analysis using CyDyeTM DIGE minimal labeling kit (GE Healthcare, Sweden), according to manufacturer’s recommendations as already described [18]. An internal standard was generated by combining equal amounts of samples and labeled with Cy2 Protein samples (50 μg) were labeled with 200 pmol of CyDye on ice in the dark for 30 min. Labeling reaction was quenced by addition of 1 μl of 10 mM lysine, and incubation was continued on ice for 10 min, in the dark. The first dimensional separation was performed at 20°C on commercial sigmoidal immobilized pH gradient strips (IPG), 18 cm long with pH range 3.5 to 10 (Pharmacia). Strips were rehydrated in 8 M urea, 2% CHAPS, 10 mM DTE, and 0.5% carrier ampholytes (Resolyte 3.5–10). The isoelectrofocusing was carried out by linearly increasing voltage from 200 to 3500 V during the first 3 hr, after which focusing was continued at 8000 V for 8 hr, as already described [33, 68–72, 75–77, 79–84]. After the first dimension separation the IPG strips were equilibrated with a solution containing 6 M urea, 30% glycerol, 2% SDS, 0.05M Tris-HCl pH 6.8, and 2% DTE for 12 min, to resolubilize proteins and reduce disulphuric bonds. The -SH groups were then blocked by substituting the DTE with 2.5% iodoacetamide in the equilibrating buffer. The focused proteins were then separated on 9–16% linear gradient polyacrylamide gels (SDS-PAGE) using a DALT six (GE Healthcare) apparatus with a constant current of 40 mA/gel at 10°C. Images were acquired with a Typhoon FLA 9500 scanner (GE Healthcare), using specific emission filters, and analyzed by the *Image* Master 2D Platinum 7 software (GE Healthcare). Protein spots showing more than 1.3 fold change in spot volume (increased for up-regulation or decreased for down-regulation), with a statistically significant ANOVA value (*p* ≤ 0.05), were considered differentially represented and further identified by MS analysis. After the acquisition, each gels were stained with ammoniacal silver nitrate, as already described [80].

### In-gel digestion and MS analysis of tryptic digests

Spots of interest were manually picked and mass spectrometric sequencing was carried out after in-gel digestion, using sequencing-grade trypsin (20 μg/vial), according to the method of Shevchenko et al. with some modifications as already described [85]. The tryptic peptide extracts were dried in a vacuum centrifuge and dissolved in 0.1% trifluoroacetic acid (TFA). Peptide mixtures were desalted by μZip-TipC18 (Millipore, MA)). The matrix, R-cyano-4-hydroxycinnamic acid (HCCA), was purchased from Sigma-Aldrich. A saturated solution of HCCA (1 μL) at 2 mg/200 μL in CH3CN/H2O (50:50 (v/v)) containing 0.1% TFA was mixed with 1 μL of peptide solution and loaded onto the MALDI target plate and left to dry. A peptide calibration standard was spotted separately onto the MALDI target plate. Mass spectra were obtained using an Ultraflex MALDI-TOF-TOF (Bruker Daltonics, Bremen, Germany) or a MALDI-TOF Voyager DE-PRO (Applied Biosystems) mass spectrometer. Peptide mass fingerprinting was compared to the theoretical masses from the Swiss-Prot or NCBI sequence databases using Mascot (http://www.matrixscience.com/). Typical search parameters were as follows: 50 ppm of mass tolerance, carbamidomethylation of cysteine residues, one missed enzymatic cleavage for trypsin, a minimum of four peptide mass hits was required for a match, methionine residues could be considered in oxidized form, no restriction was placed on the isoelectric point of the protein, and a protein mass range from 5 to 100 kDa was allowed.

### Transmission electron microscopy (TEM) analysis

Cells were grown to confluence in a 12-well plate and treated with appropriate concentrations of both AgNPs-EPS for 24 h, washed in 0.075 M phosphate buffer pH 6.9 and immediately fixed in a solution of 3% glutaraldehyde in cacodylate buffer 0,066M pH 7.2 for 30 min as already described [72]. Then the cells were dehydrated using increasing concentrations of ethanol (10, 30, 50, 70 and 100%) at room temperature and gradually infiltrated in Spurr’s low viscosity embedding resin and polymerized at 70°C for 8 h. After polymerization, specimens were sectioned with an ultramicrotome (model LKB III) using a diamond knife. The thin sections were collected on copper grids, stained for 10 min in 2% uranyl acetate and 5 min in lead citrate and then they were observed by Transmission Electron Microscopy (PHILIPS 268 Morgagni, FEI ) operating under standard conditions.

### Fluorescence spectra of DNA

The emission-excitation (EEM) spectra of DNA were recorded by Luminescent Spectrometer (Perkin Elmer LS 55). The instrument was equipped with a Xenon discharge lamp equivalent to 20KW for 8 µs duration pulse, wide at half eight minor 10µs with excitation slit 5 nm and emission slit 5 nm. The extracted DNA was suspended in 1 ml milliQ and transferred in cuvette for fluorescence analyses. The EEM scan spectra were measured by scanning simultaneously both the excitation (E_ex_), which ranged from 200 to 320 nm, and the emission (E_em_), which ranged from 200–700 nm. The EEM contour maps were obtained by original equipped computer software. The relative fluorescence units (RFU) of peaks were also determined.

### Statistical analysis

Results are presented as means ± standard error. Data were analysed using Student’s *t*-test. *p* < 0.05 was considered significant; *p* < 0.01 was considered highly significant and *p* < 0.001 was considered very highly significant.

## SUPPLEMENTARY MATERIALS TABLES






