# Supplementary material for: Anticancer activity of biogenerated silver nanoparticles: an integrated proteomic investigation
Source: Oncotarget. 2017 Dec 23;9(11):9685–705. doi: 10.18632/oncotarget.23859 (PMC5839394; doi:10.18632/oncotarget.23859)
Supplement: Supplementary file 1 [file oncotarget-09-9685-s001.pdf]

Anticancer activity of biogenerated silver nanoparticles: an integrated proteomic investigation

SUPPLEMENTARY MATERIALS

Supplementary Table 1: List of differentially regulated proteins reporting spot identity, fold change, percentage of volume spots, ANOVA values, calculated by the software. See Supplementary_Table_1

Supplementary Table 2: List of the identified proteins reporting MS data informations. See Supplementary_Table_2
